# Striking the right balance: co-designing the Health4Me healthy lifestyle digital health intervention with adolescents

**DOI:** 10.1186/s40900-023-00524-4

**Published:** 2023-12-07

**Authors:** Rebecca Raeside, Allyson Todd, Sara Wardak, Lauren Gardner, Katrina E. Champion, Melissa Kang, Seema Mihrshahi, Katharine Steinbeck, Julie Redfern, Stephanie R. Partridge, The Health Advisory Panel for Youth at the University of Sydney (HAPYUS), Radhika Valanju, Meera Barani, Dominik Mautner, Imeelya Al Hadaya, Alexi Cross, Melani Gunawardana, Ava Lambie, Emily McMahon, Arnav Narula, Bowen Ren, Dominique Rose, Aviral Sharda, Alexander Sinnett, Azman Tanvir, Fulin Yan, The Health4Me Team, Karice Hyun, Maree L. Hackett, Gemma Figtree, Robyn Gallagher, Karen Spielman, Sarah Maguire, Kyra A. Sim, Tim Usherwood, Charlotte Hepse, John Skinner, Liliana Laranjo, Kathryn Williams, Danielle Castles

**Affiliations:** 1 Engagement and Co-design Research Hub, School of Health Sciences, Faculty of Medicine and Health, The University of Sydney, Sydney, NSW Australia; 2 The Matilda Centre for Research in Mental Health and Substance Use, University of Sydney, Sydney, NSW Australia; 3 General Practice Clinical School, Sydney Medical School, Faculty of Medicine and Health, University of Sydney, Sydney, NSW Australia; 4 Department of Health Sciences, Faculty of Medicine, Health and Human Sciences, Macquarie University, Sydney, NSW Australia; 5 Specialty of Child and Adolescent Health, Westmead Clinical School, Faculty of Medicine and Health, University of Sydney, Westmead, NSW Australia; 6 The George Institute for Global Health, University of New South Wales, Kensington, NSW Australia; 7 Charles Perkins Centre, University of Sydney, Sydney, NSW Australia

**Keywords:** Adolescent, Co-design, Text-message, Behavior change, Intervention, Prevention, Digital health

## Abstract

**Background:**

Adolescents are navigating a period of rapid growth and development within an era of digitalization. Mobile phone ownership among adolescents is nearly ubiquitous, and this provides an opportunity to harness text messaging to promote a healthy lifestyle and reduce chronic disease risk factors. Inclusion of adolescents throughout the design process has been recognized as essential for engagement and future implementation of such interventions. This study aimed to co-design a bank of text messages to promote a healthy lifestyle which are useful, acceptable, and engaging for adolescents aged 12–18 years old.

**Methods:**

Iterative, mixed-methods design with consumer partnership. Co-design occurred over three stages: text message development, text message review and final refinement and testing. The text message development included literature searches and consumer partnership with an established youth advisory group (n = 16). Participants who gave e-consent participated in text message review. Demographic characteristics were collected, and quantitative surveys were distributed to adolescents (n = up to 50) and health professionals (n = up to 30), who rated text message content for understanding, usefulness and appropriateness (total score out of 15). Final refinement was completed by the research team to edit or remove messages which had low scores and to assess readability and interactivity of the text messages.

**Results:**

The Heath Advisory Panel for Youth at the University of Sydney (HAPYUS) identified the top six lifestyle health issues for young people today in relation to chronic disease prevention, which became the key content areas for the text message bank and drafted new text messages. Following text message development, 218 messages were available for review. Adolescents (n = 18, mean age 16.3 [SD 1.4]) and healthcare professionals (n = 16) reviewed the text messages. On average, all reviewers found that the text messages were easy to understand (mean = 13.4/15) and useful (mean = 12.7/15). Based on scoring and open ended-feedback, 91 text messages were edited and 42 deleted. The final text message bank included 131 text messages. The overall program is suitable for a seventh-grade reading level, and interactive.

**Conclusions:**

This study describes the process of effectively engaging adolescents to co-design a text message bank intervention, which are useful, acceptable and engaging for an adolescent audience. The effectiveness of the co-designed text message bank is currently being tested in the Health4Me RCT.

**Supplementary Information:**

The online version contains supplementary material available at 10.1186/s40900-023-00524-4.

## Abstract (plain language summary)

Today’s adolescents are growing and developing through a period of increased technology use. Most adolescents have access to a mobile phone which can be used to deliver healthy lifestyle information to them through text messages. However, it is important that researchers engage with adolescents as collaborators to develop any information which may be sent to them, to ensure that it is acceptable and engaging. We aimed to co-design a bank of text messages to promote a healthy lifestyle which are useful, acceptable and engaging for adolescents 12–18 years old. We engaged with 16 adolescents from an established youth advisory group who advised on top health issues they face today in terms of leading a healthy lifestyle, which became key content areas for the text message program. They also drafted text messages around these content areas (218 total). Next, the text messages underwent review with 34 adolescents and healthcare professionals to ensure they were useful, easy to understand and appropriate. After the review, the research team edited, deleted and replaced text messages which did not score well. This resulted in a text message bank to promote a healthy lifestyle with 131 text messages which were useful, acceptable and engaging for adolescents 12–18 years old.

**Supplementary Information:**

The online version contains supplementary material available at 10.1186/s40900-023-00524-4.

## Introduction

Adolescence is a period of significant biological and neurocognitive growth, emotional development, and social change. Some of the biggest challenges facing adolescents in Australia today are unhealthy diets, physical inactivity, excessive screen time, sedentary behavior and poor sleep, all of which have been linked to poor physical and mental health, including overweight/obesity, anxiety, depression and psychological distress [1]. Today’s adolescents are navigating this crucial period of life amid the rapid wave of digitalization [2]. Their engagement with digital media, such as smartphones, the internet, and social media, exposes them to a diverse spectrum of advantages and challenges that can impact their physical and mental health [3]. Emerging research demonstrates that there is potential for digital health lifestyle interventions to improve the physical and mental health of adolescents [4, 5]. It is vital that health services provide solutions which meet the needs of adolescents through engaging digital media and equipping them with information and tools to combat the challenges they face. This will assist in the development of healthy lifestyle behaviors which they can carry throughout life and reduce future risk of non-communicable diseases, including cardiovascular disease, type 2 diabetes and mental ill-health.

Adolescent mobile phone ownership has grown internationally, and 9 in 10 Australian teenagers have a mobile phone [6]. With that comes the opportunity to harness these devices to provide information and tools to adolescents to improve their lifestyle health behaviors through mobile applications, social media and text messaging. Though social media is popular amongst adolescents, a previous meta-analyses of social media interventions for diet and exercise behaviours showed no significant differences between groups and had low levels of participation [7]. Furthermore, there are challenges to running these interventions including the need to separate control for the effects of existing social media structures [8] and how to effectively evaluate the intervention [9]. Text message strategies are the most accessible, as these are low-cost to send, free to receive and do not require an internet connection [10]. It is important to ensure that digital health tools are not driving digital exclusion and health inequalities due to limited access or ability of recipients to use them, or cost [11]. Additionally, text messaging is the preferred form of communication for adolescents [12]. Its use may overcome barriers to preventive health care, including cost of visits, transport to and from services and opening hours [13]. Previous research has shown that texting can help adolescents navigate key developmental challenges including establishing autonomy, facilitating connection with peers and self-identity [14]. However, there is limited research on text message programs aimed at promoting health and preventing chronic disease among otherwise healthy adolescents through the improvement of lifestyle health behaviors.

It is a human right that adolescents are engaged in research and policy development which impacts their own health [15]. Furthermore, the World Health Organization recommends that young people should be included in every stage of the research process when developing digital health interventions, and that their engagement should be sustained and meaningful [16]. Traditional adolescent engagement in research has been tokenistic. A recent scoping review showed that just 11% of all research on obesity prevention engaged adolescents in all five stages of the research cycle (identification of topic, design or development, conduct, analyses and dissemination) and only 9% of studies involved an adolescent-led approach [17]. Adolescents need to be leaders in decisions about their health for impacts to be seen now and for generations to come. Yet for this to occur, a shift in power is necessary.

Co-design of digital health interventions with adolescents is a potential solution, a process in which active collaboration occurs between stakeholders (adolescents, healthcare professionals, researchers) in designing solutions to a pre-specified problem [18]. Considering digital health interventions are increasing in popularity for health-related behavior support, it is essential that they are co-designed with adolescents. Though many frameworks for co-design are available, no existing frameworks are specific to adolescents, and it has been suggested that locally relevant co-design activities may be more useful than a one-size-fits-all framework [19]. There must be an equal relationship, where consumers can co-lead the development, design and implementation process to tailor interventions to their lived experience around their values, goals and development. A previous systematic review found four overarching themes which affect engagement and enrolment in digital health interventions including: personal agency and motivation, personal life and values, the approach used for engagement and recruitment and the quality of the intervention itself [20]. Therefore, digital health interventions which do not take co-design into account can lead to disengagement and enrolment issues that adversely impact the implementation phase [21].

The Health4Me Study is a randomized controlled trial aiming to determine the effectiveness of a co-designed 6-month text message program to improve adolescents’ healthy lifestyle behaviours. In brief, the intervention group receive a 6-month text message program (4–5 text messages per week) with optional monthly health counselling. The trial is currently in the recruitment phase and the full study protocol has been previously published [22]. The aim of this study is to co-design a bank of text messages that promote a healthy lifestyle which are useful, acceptable, and engaging for adolescents aged 12–18 years. The co-designed message bank will be tested for effectiveness in the Health4Me study.

## Methods

### Study design

This study employed an iterative, mixed-methods study design, with consumer partnership to co-design the Health4Me text message bank. This process involved adolescents aged 12–18 years and a variety of healthcare professionals and researchers (across fields of public health, behaviour change, physical activity, nutrition and psychology). The co-design occurred over three stages: (i) text message content development, including literature search and consumer partnership (ii) text message review following a previously published process [23, 24] and (iii) final refinement and programming. This study was conducted between July 2022–January 2023. Ethics approval was obtained from the University of Sydney Human Research Ethics Committee (approval number: 2022/402).

### Text message development

The aim of this stage was to develop the initial bank of text messages. Several strategies were employed to develop the text message program including (i) an initial search of published literature to identify key behavior change techniques to underpin the text messages which may be effective among adolescents and (ii) online collaboration via monthly meetings over 12 months and a 1.5-h workshop with an established youth advisory group (The Health Advisory Panel for Youth at the University of Sydney; HAPYUS). HAPYUS is comprised of 16 members (aged 13–18 years) from across the state of New South Wales, Australia and serve a 12-month term as collaborators with the research team. The youth advisory group is informed by Youth Participatory Action Research principles [25], (i) inquiry based, topics of discussion were grounded in youth advisors lived experience and concerns related to chronic disease prevention; (ii) participatory, youth advisors are collaborators in the research process; and (iii) transformative, youth advisors will actively intervene to change research to improve the lives of youth and their communities from the negative impacts of chronic diseases.. Within the structure of the youth advisory group, adolescents were provided with opportunities to co-lead and contribute to chronic disease prevention research projects. The lead author presented to HAPYUS about the Health4Me Study to give them background and context to what they were being asked to contribute to, as some elements were predetermined. What was predetermined is that the intervention would be delivered via text messages and that it would run for 6-months. Within the capability of the text message delivery software, text messages can be scheduled to be sent at different times and on different days. HAPYUS were first asked to identify the top lifestyle health issues for young people, which became the key content areas for the intervention. At the in-person meeting they were then asked to draft text messages for adolescents 12–18 years old who have no chronic medical conditions, determine the frequency of messages to be sent per week and comment on the time of day that they would like to receive text messages. A young person research assistant (SW) worked with HAPYUS to collate the drafted text messages.

### Text message review

The aim of this stage was to test the content of the draft text message bank using a mixed-methods survey based on a previous published process [23, 24]. The text messages developed in the first stage were combined with an existing bank of 107 co-designed text messages [24, 26]. Each text message was rated six times, three times by adolescents from the public and three times by health professionals or researchers. Each participant rated 40 text messages, and adolescents were compensated for their time with a $30AUD gift voucher.

#### Participants

Adolescents were included if they (i) were aged 12–18 years; (ii) provided informed e-consent (or with their parents or guardians consent if ≤ 14 years old). Adolescents were excluded if they (i) had a medical condition that precluded informed consent or their ability to comply with the study protocol; (ii) were unable to read English at a 7^th^ grade level. Adolescents who were part of the youth advisory group were unable to take part in this process. Professionals were included if they (i) were multidisciplinary clinical and research experts including, but not limited to dietitians, physiotherapists, psychologists, exercise physiologists, general practitioners, behavioral science experts and public health researchers; (ii) provided informed e-consent. Recruitment of adolescents occurred through convenience sampling via mailing lists of young people who consented to being contacted for research participation by the research team, and professionals were recruited through known networks to the research team. Adolescents and professionals were invited via email, directed to read the participant information sheet and provide informed e-consent if they wished to take part.

#### Data collection and feedback

First, demographic characteristics were collected for adolescents and healthcare professionals who had experience working with adolescents. Demographics collected from adolescents included age, gender, language spoken most at home, education and postcode. Postcode was used to determine participants’ Index of Relative Socio-economic Advantage and Disadvantage (IRSAD), which codes postcodes into quintiles from 1 (most-disadvantaged area) to 5 (least disadvantaged area) [27]. Demographics from professionals included age, gender and area of expertise. Text messages were placed into surveys comprised of 20 text messages, with some surveys about one topic and some about mixed topics. This helped to ensure that professionals were reviewing text messages most relevant to their area of expertise. For each message, the survey comprised two questions around understanding (able to comprehend what the message is saying) and usefulness (able to be practically used or used in several ways) on a 5-point Likert scale (response options: 1. Strongly disagree, 2. Disagree, 3. Neutral, 4. Agree, 5. Strongly agree), one question around age appropriateness (response options: 12–14 years of age, 15–16 years of age, 17–18 years of age) and a final open-ended question where suggestions for improvement could be made.

#### Analysis

Scores (15 indicating the best score) for understanding and usefulness were calculated separately for adolescents and professionals by summing the scores of each reviewer (5 points each, 5 indicating the best score). Any text messages which scored fewer than 12 points across any category were edited or excluded. All open-ended feedback was summarized, and concerns or suggestions were adhered to. An updated text message bank was then available for final checks and text message delivery system testing.

### Final refinement and testing

The aim of this stage was to consolidate the findings of the development and review stages to ensure the final text message bank was ready for effectiveness testing in a six-month healthy lifestyle intervention trial. Firstly, text messages were added back into their respective categories to ensure that there were adequate text messages in each. Secondly, the readability of each text message was calculated using the Flesch-Kincaid readability score [28]. This score represents an approximate education level an individual would need to have to understand the reading material considering the number of syllables per word and number of words per sentence. Finally, the number of hyperlinks and two-way messages within the program was calculated to understand the interactivity of the program. All text messages were reviewed one final time by the young person research assistant and programmed for a test delivery into the text message software.

## Results

### Text message content development

Multiple studies were identified in the literature search which supported the use of different behavior change techniques and theories. Rose and colleagues conducted a systematic review of digital interventions for improving the diet and physical activity behaviors of adolescents and found that significant behavior change was seen when education, goal setting, self-monitoring and parental involvement were included [4]. Martin and colleagues conducted a systematic review of effective behavior change techniques for prevention or management of childhood obesity and found that only ‘prompting generalization of a target behavior’ was effective for causing behavior change for obesity prevention [29]. For this study, we used the CALO-RE Taxonomy of behaviour change techniques [30], which is based on Abraham and Michie’s initial taxonomy [31], with a specific focus on physical activity and healthy eating. The following behaviour change techniques derived from the literature search were applied to the initial text messages: goal setting, action planning, prompt generalization of a target behavior, provide instruction on how to perform the behavior, plan social support and prompt self-talk.

Through online collaboration, HAPYUS created a mind-map to identify the top lifestyle health issues for young people today in relation to chronic disease prevention. They identified six key issues: unbalanced nutritional intake, physical inactivity, mental health concerns, body image, rise of social media and climate change [32]. These became the six key content areas for the Health4Me text message program. In July 2022, an in-person workshop was held with 9/16 HAPYUS members able to attend. During the 1.5-h workshop, 105 new text messages were developed in alignment with the six key content areas. In addition, linking adolescents to primary care services was an overall theme which the research team deemed as important for preventive health care and therefore six messages were added (one per month). Therefore, a total of 111 new text messages were available. Post-workshop, the research team edited these text messages briefly to ensure that they were evidence-based and included behaviour change techniques. During the workshop, a consensus was reached on the timing and frequency of text messages. The adolescents revealed that receiving messages before and after school would be acceptable, but to consider the content and deliver at the appropriate time (e.g., deliver text messages about sleep in the evening). Furthermore, it was decided that messages should be less frequent at the start of the program and increase as the program continues. Taken together, a program where adolescents receive four text messages per week at the start and increases to five per week, is optimal. Patient and public involvement in the study is outlined in Additional File 1.

### Text message review

A total of 218 text messages were available for review, combining the 111 new text messages developed and 107 existing co-designed text messages. The bank of 218 text messages were reviewed by 34 participants (Table 1). Adolescents (n = 18) had a mean age of 16.3 years (SD 1.4), with 8 male, 8 female and 2 non-binary/gender diverse participants, who mostly spoke English at home (17/18) and were predominantly from IRSAD quintiles four and five, corresponding to least disadvantaged areas (10/17). Sixteen professionals participated who were predominantly 30–39 years of age (9/16), and female (12/16).

**Table 1:** Participant characteristics (n = 34)

Adolescents	N = 18
Age (mean years ± SD)	16.3 ± 1.4
Gender	
Female	8
Male	8
Non-binary/gender diverse	2
Language spoken at home^a^	
English	17
Current high school student	
Years 7–8	1
Years 9–10	4
Years 11–12	12
Not attending school	1
IRSAD quintile	
1 (least disadvantaged)	7
2	3
3	5
4	1
5 (most disadvantaged)	2

Professionals	N = 16
Age	
18–29	3
30–39	9
40–49	2
50–59	2
Gender	
Female	12
Male	4
Area of Expertise^b^	
Physical activity	2
Nutrition and diet	6
Medicine	3
Public Health	8
Prevention	8
Behaviour change	6
Psychology	3
Other	1

*SD* Standard deviation, *IRSAD* Index of Relative Socio-economic Advantage and Disadvantage

^a^Other language spoken at home was Hindu

^b^Professionals could pick more than one area of expertise

On average, expert reviewers found that the text messages were easy to understand (13.7/15) and useful (13.3/15), as did adolescent reviewers (13.2/15 for understanding and 12.1/15 for useful). A total of 96 of the 218 text messages scored less than 12 by at least one participant. Based on scoring and open-ended feedback, 91 text messages were edited and 42 were deleted, leaving 176 messages from the review process. Text messages which scored highly gave practical examples, contained links, and focused on elements other than just physical health. Common edits to text messages were wording changes to make age-appropriate, sentence restructure, and concept definition. Table 2 displays examples of text messages which were included, edited, replaced, and deleted.

**Table 2:** Example text messages and scoring for text message inclusion, exclusion, replacement and editing

	Original message	Scores	Comments	Edited message
Edited	Hey you, yes you! Reminding you to take a break from the screens—your eyes need a rest. How about 15 min outside to get some fresh air too!	P^a^: 15/15 for understanding, 15/15 for usefulness A^b^: 14/15 for understanding, 13/15 for usefulness	A1: The start bit is a little cringey A2: Good message	Hi [pref_name], reminding you to take a break from the screens—your eyes need a rest. How about 15 min outside to get some fresh air too!
Edited	We understand its really tough to manage your time when all of your assessments are due at the same time! It can be rough. Hang in there [pref_name]. You're doing great!	P: 12/15 for understanding, < 12/15 for usefulness A: 15/15 for understanding, 14/15 for usefulness	P: A link to time-management or study skills resources could be helpful A: Maybe provide some solution to this issue? I know this is a source of stress for a lot of students, myself included	We understand its really tough to manage your time when all of your assessments are due at the same time! It can be rough. Hang in there [pref_name]. You're doing great! Learn some tips here: https://au.reachout.com/articles/how-to-manage-your-time
Included	We can help the environment and improve our health at the same time! A win–win, text back if you have done any of the following this week: (a) used a reusable water bottle; (b) ate less processed foods; (c) used reusable food containers, or (d) all of the above!	P: 14/15 for understanding, 15/15 for usefulness A: 14/15 for understanding, 14/15 for usefulness	P: Great, loved this message! Can help the participant feel good about themselves if they have managed to do some of the options listed: A: can provide opportunity for interaction for those who havent done the options	No change
Included	Complimenting your friends or family members on an aspect of their personality or behaviour, rather than on their appearance can brighten their mood—it can also make you happier knowing you made someone smile!	P: 15/15 for understanding, 15/15 for usefulness A: 14/15 for understanding, 15/15 for usefulness	P: love it	No change
Excluded	What if soy milk is just regular milk introducing itself in Spanish? Hola! Soooy funny. Soy milk isn't Spanish. It’s made from soybeans, which are naturally low in calcium. If you drink soy milk, make sure to grab one with extra calcium added in so you can build strong bones!	P: < 12/15 for understanding, 14/15 for usefulness A: < 12/15 for understanding, < 12/15 for usefulness	P: I love this	Deleted
Excluded	I hear bouldering's really popular nowadays—why not try it at a rock climbing centre and see if you like it, [pref_name]?	P: 14/15 for understanding, 13/15 for usefulness A: A: < 12/15 for understanding, < 12/15 for usefulness	A: Why bouldering? Is there an actual purpose to why you said that? Why not something more approachable for a beginner—because bouldering is not	Deleted
Replaced	Leafy greens like spinach are packed with protein, so they’re great for building muscles. How about in a blue smoothie? Just blend 1 cup frozen blueberries + 1 cup milk + 1 tsp honey + 2 cups of baby spinach (you won't taste it!)—[centre_name]	3/6 voted not appropriate for 12–14-year-olds	N/A	Replaced in 12–14 years old message stream with message below
Replaced	Did you know elephants and gorillas are some of the strongest animals on the planet and are also herbivores? Remember to keep greens in your diet to stay strong!	2/6 voted not appropriate for 17–18-year-olds 1/6 voted not appropriate for 15–16-year-olds	N/A	Replaced in 15–18 years old message stream with message above

^a^Professional reviewer

^b^Adolescent reviewer

Following scoring and editing, text messages were placed into their respective topic categories to determine how many were required. Similar text messages were combined and those with lower scores were removed to create the final text message bank. When assessing the age-appropriateness of text messages, 28 were deemed inappropriate for 12–14-year-old participants, nine were inappropriate for 15–16-year-old participants and eight were inappropriate for 17–18-year-old participants. Considering the split and difference in age-appropriate text messages, two streams of text messages were created, one stream for adolescents aged 12–14 years and one for adolescents aged 15–18 years. Text messages deemed inappropriate were deleted or replaced with an age-appropriate message in their respective programs. An overview of text message numbers for each category is available in Table 3.

**Table 3:** Overview of number of text messages through the text message review process

Topic of text message content	Total number of text messages^a^	Number of text messages post-review	Number of text messages in Health4Me program
Nutrition	56	47	32
Physical Activity	41	30	26
Mental Health	50	41	27
Body Image	21	18	12
Media	14	11	10
Climate	30	23	10
Primary Care	6	6	6
Total:	218	176	123^b^

^a^Combined from the TEXTBITES text message program and created through the co-design workshop

^b^Total text message bank is 131 messages once the introductory and final messages are added, along with 6 messages to encourage communication with health counsellor

### Final refinement and testing

**Fig. 1:**
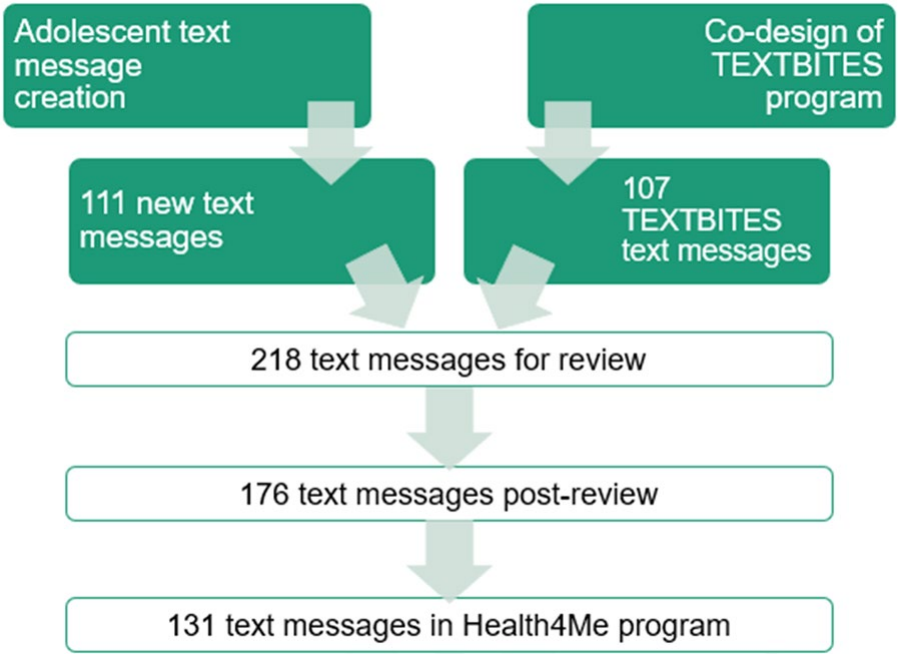
Flow of text messages through the text message review process

The final text message bank comprised 131 messages (Fig. 1); frequency of message topics was nutrition n = 32, physical activity n = 26, mental health n = 27, body image n = 12, media n = 10, climate n = 10 and primary care n = 6. In addition, there was an introductory and final text message (n = 2), and six messages encouraging communication with the health counsellor.

Almost half of the text messages simultaneously addressed two behaviour change techniques. Text messages addressed goal setting (n = 56), action planning (n = 25), prompt generalization of a target behavior (n = 17), provide instruction on how to perform the behavior (n = 43), plan social support (n = 14) and prompt self-talk (n = 9). The average Flesch Kinkaid readability score for the Health4Me program was 75.4 (SD 12.9), indicating that the overall program is fairly easy to read and suitable for a seventh-grade (12–13 years old) reading level. Most text messages (n = 85) were rated very easy to read to fairly easy to read (5th–7th grade reading level; 11–13 years of age), 30 messages were in plain English (8 to 9th grade reading level; 13–15 years of age), 16 messages scored fairly difficult to read (10–12th grade reading level; 16–18 years of age). Small punctuation edits were made to these messages, where possible, to reduce the scores. There were six messages which encouraged interaction with the health counsellor with one sent each month of the program. Twenty-nine text messages encouraged two-way communication, and 47 text messages contained links to external websites (e.g., recipe or exercise ideas, further information, referring to available services). Only three text messages differed between the 12–14 and 15–18-year-old text message streams. A final delivery system check was completed with 55 text messages sent over 14 days to 5 members of the research team, with all successfully delivered, and with 15 replies from the research team noted in the system.

### The Health4Me intervention

Based on results of the co-design process, some specific elements were decided upon for the Health4Me intervention. For the first 13 weeks of the intervention participants receive four text messages per week, which increases to five text messages per week in the last 13 weeks. Each week participants in the intervention group will receive one text message on nutrition, one text message on physical activity, and a mix of two to three text messages from other topics. Once enrolled in the correct text message stream for their age, all participants will receive the same text messages. Text messages will be personalized with the participants preferred name. Engagement with the text message program will be assessed through text message data and a study-specific user feedback and satisfaction questionnaire.

## Discussion

This study describes a novel way to co-design a healthy lifestyle text message intervention which is useful, engaging and acceptable for adolescents. A unique aspect of this study is putting adolescents at the forefront to advise on content areas which they consider are most influential on their lifestyle health behaviors in a modern context. Through an iterative co-design process, a bank of 131 evidence-based text messages which are easy to understand and acceptable to adolescents were developed. This study engaged a youth advisory group (HAPYUS), adolescents from across Australia, and health professionals to incorporate contemporary issues as the key content areas of the text message bank. The intervention is grounded in behaviour change techniques and through the text message review, messages which scored highly gave practical examples, contained links and focused on elements other than just physical health.

Traditionally, preventive health for adolescents focuses on high-risk behaviors including sexual activity, alcohol and substance use and smoking, in addition to physical activity and nutrition behaviors [33]. However, adolescents from the youth advisory group identified contemporary influences which are vital to consider in a preventive health context. including the rise of social media, body image, mental health and climate change, in addition to nutrition and physical activity [32]. Research also supports these issues as important within an adolescent health context. Emerging research has demonstrated a ‘clustering effect’, where adolescents who engage in multiple risky lifestyle behaviors have more symptoms of mental illness [34, 35]. The average adolescent today spends 1.6 h per day on social media [36], which is consistently and positively associated with negative body image [37], and the environment was considered the most important issue to 51% of young people across Australia in 2022 [38]. In addition, previous reviews have shown that text message interventions are effective in changing preventive health behaviors [39]. Our co-design process has demonstrated that preventive health care for today’s adolescents requires a broader scope and provides direction to address emerging public health issues.

Our research also demonstrated the input required to effectively work with adolescents as co-researchers. Previous preventive health interventions have been tested amongst adolescents, but evidence is lacking to support translation into effective interventions which have been implemented into health services [40]. This study has a strong focus on engaging adolescents to co-design the intervention to produce downstream benefits. Involving adolescents in research about them allows for benefits to the research, the adolescents and to the community [41]. Thus far, adolescents have been involved in three phases of the research, namely: identification of topic, design or development and conduct. Specific to this research, the intervention is co-led by adolescents who identified the key issues for chronic disease prevention [32] and drafted text messages around these issues. Adolescents also advised on recruitment materials to ensure they were appropriate. This potentially has further benefits to the ongoing research, by increasing recruitment and retention of participants and the effectiveness for the intervention in this study. We will conduct a thorough process evaluation to understand these factors when the trial concludes. A separate study evaluated the leadership and life skills of adolescents who were involved in co-designing the intervention (HAPYUS) and found that they improved over 12-months [42]. Finally, there are possible benefits to the wider community, including increased awareness of relevant health issues for adolescents, as has been seen in previous research [43]. By shifting the power to adolescents to co-design the Health4Me intervention, the potential benefits may accelerate the implementation of this research due to its relevance among an adolescent population [41].

There are some more specific learnings from the text message review process, including the importance of striking the right balance in the content of the text messages. In many cases, there was conflicting comments between professionals and adolescents, and even in some instances between two different groups of adolescents. For example, messages with humor were often seen as ‘trying too hard’, whereas other adolescents were calling for more humor in the messages. In these cases, it is important to remember who the program is being designed for and to preference the comments of adolescents over those made by professionals, except in the case of evidence-based information. Previous research has shown that adolescents seek lifestyle health information which is credible [44]. This finding was mirrored in this study where messages which scored highly contained links to reputable sources online, and common edits to messages included defining concepts for greater understanding, which often included adding hyperlinks to more information. The review process confirmed the findings of another study, where adolescents preferred messages which referred to them and recommended specific and achievable behaviors [45]. The current study extended this concept, where higher-scoring messages contained practical examples and focused on elements other than just physical health that confirmed experiences of being a young person today.

It is acknowledged that whether these carefully curated messages will result in changes in nutrition and physical activity behaviors is not yet known. The effectiveness of the text message program is currently being tested in a randomized controlled trial (RCT) [22]. The behavior change techniques selected were based on two systematic reviews [4, 29], one of which suggested that parental involvement was a key technique to eliciting significant behavior change. The delivery of this intervention directly to adolescents means that parental involvement could not be addressed, and parental involvement also changes with age as autonomy evolves during adolescence. To acknowledge external supports, we chose to include the behaviour change technique ‘plan social support’, that has been found useful in interventions for nutrition and physical activity behaviors [46]. Furthermore, adolescents who receive the intervention will also have the option to engage with a health counsellor to provide further support for behavior change using motivational interviewing techniques [47], which have already shown promise in adolescent obesity prevention programs [48, 49]. A detailed evaluation to understand acceptance and engagement with the program through text message software data (delivery, responses, retention rate), quantitative feedback surveys completed by all intervention participants and virtual focus groups with a smaller subset of participants will be part of the RCT [22]. Though adolescents were not consulted on which behaviour change techniques to employ in this study, there is an opportunity for future research to engage with adolescents on this topic to combine the published evidence and views of the intended audience to drive effectiveness and engagement.

There are limitations of this study. Firstly, due to the nature of research and funding cycles, the grant to fund this work was awarded and the youth advisory group set up afterwards. This means that the concept of the project as a text message intervention had already been decided upon before the youth advisory group was assembled. True co-design includes involving consumers through all stages of the development, including the study design [18]. The youth advisory group did not raise any issues with the intervention delivery method, suggesting that the planned research method was acceptable. Secondly, due to recruitment of adolescents for the text message review through convenience sampling, a small number of younger adolescents in Years 7 and 8 (ages 12–13 years) reviewed the text messages, and only one who spoke a language other than English at home. Therefore, there may be a bias for the text message content to be geared toward adolescents aged 15 years and above and potentially be incompatible with culturally and linguistically diverse adolescents and those who are from areas of socioeconomic disadvantage. We have taken steps to mitigate these effects. Firstly, adolescents > 14 years old are able to consent themselves into the randomized controlled trial. This removes the need for parent/guardian consent for this age group, where the parent/guardian may not have proficient English language skills to be able to provide consent but the adolescent does. Secondly, by conducting the Flesch-Kincaid scoring for readability, we ensured the overall text message bank is at a seventh-grade reading level for those who regularly speak and read English.

## Conclusion

This study describes the intensive co-design a healthy lifestyle text message intervention, comprised of 131 text messages which are useful, acceptable and engaging for adolescents 12–18 years old. The bank of text messages was developed based on prior research, effective behaviour change techniques for improving nutrition and physical activity behaviours and through adolescent identification of the top lifestyle health issues related to chronic disease prevention (A). This co-designed text message bank is currently being tested for effectiveness in the Health4Me Study. If effective, text message interventions hold potential to be an accessible and affordable method of delivering preventive health information to adolescents to reduce risk of future chronic disease development.

### Supplementary Information

**Additional file 1.** GRIPP2 Short Form checklist.

## Data Availability

Data sharing is not applicable to this article as no datasets were generated or analysed during the current study.

## Abbreviations

HAPYUS: The Health Advisory Panel for Youth at the University of Sydney
IRSAD: Index of Relative Socio-economic Advantage and Disadvantage
RCT: Randomized controlled trial
