# Supplementary material for: Striking the right balance: co-designing the Health4Me healthy lifestyle digital health intervention with adolescents
Source: Res Involv Engagem. 2023 Dec 7;9:114. doi: 10.1186/s40900-023-00524-4 (PMC10702116; doi:10.1186/s40900-023-00524-4)
Supplement: Supplementary file 1 — Additional file 1. GRIPP2 Short Form checklist. [file 40900_2023_524_MOESM1_ESM.docx]

**Additional File 1: GRIPP2 Short Form checklist**

| **Section and Topic** | **Item** | **Reported on page no.** |
| --- | --- | --- |
| 1. **Aim** | Report the aim of PPI^a^ in the study | p.6 |
| 1. **Methods** | Provide a clear description of the methods used for PPI in the study | p.7-8 |
| 1. **Study results** | Outcomes—Report the results of PPI in the study, including both positive and negative outcomes | p.11-12 |
| 1. **Discussion and conclusions** | Outcomes—Comment on the extent to which PPI influenced the study overall. Describe positive and negative effects | p.16-18 |
| 1. **Reflections/critical perspective** | Comment critically on the study, reflecting on the things that went well and those that did not, so others can learn from this experience | p.19-20 |

*^a^PPI* patient and public involvement
